# Clinical outcomes of CyberKnife stereotactic radiotherapy for localized prostate cancer: comparison of 35 Gy in 5 fractions and 36 Gy in 4 fractions

**DOI:** 10.1093/jrr/rrag017

**Published:** 2026-04-14

**Authors:** Jiyoon Lim, Yoon Y Jo, Sumin Lee, Yeon J Kim

**Affiliations:** Department of Radiation Oncology, Asan Medical Center, University of Ulsan College of Medicine, 88 Olympic-ro 43-gil, Songpa-gu, Seoul 05505, South Korea; Department of Radiation Oncology, Yeungnam University Medical Center, 170 Hyeonchung-ro, Nam-gu, Daegu 42415, South Korea; Department of Radiation Oncology, Kangwon National University Hospital, 156 Baengnyeong-ro, Chuncheon-si, Gangwon-do 24289, South Korea; Department of Radiation Oncology, Asan Medical Center, University of Ulsan College of Medicine, 88 Olympic-ro 43-gil, Songpa-gu, Seoul 05505, South Korea

**Keywords:** CyberKnife, stereotactic body radiation therapy, prostate cancer, dose escalation, toxicity

## Abstract

This retrospective study evaluated the clinical outcomes of two CyberKnife (CK) stereotactic body radiation therapy (SBRT) regimens in patients with localized prostate cancer (LPC). A total of 249 patients were treated, with 123 receiving 35 Gy in 5 fractions and 126 receiving 36 Gy in 4 fractions, with a median follow-up of 50 months. The primary endpoint was the incidence of grade ≥2 genitourinary (GU) and gastrointestinal (GI) toxicities, assessed using CTCAE Version 5.0. Secondary endpoints included 3-year biochemical recurrence-free survival (BCRFS), locoregional recurrence-free survival (LRRFS) and distant metastasis-free survival (DMFS). To address baseline imbalances between groups, inverse probability of treatment weighting (IPTW) was applied. Acute grade 2 GU toxicities occurred in 19 patients (7.6%) and chronic grade 2 in 15 patients (6.0%). Acute and chronic grade 2 GI toxicities were rare, occurring in 3 (1.2%) and 2 (0.8%) patients, respectively. There were no significant differences in cumulative GU or GI toxicity rates between fractionation regimens either before or after IPTW adjustment. After IPTW, the 3-year BCRFS was 96.5% in the 5-fraction group and 96.8% in the 4-fraction groups (*P* = 0.571). The 3-year LRRFS rates were similarly favorable (99.2% vs. 100%). The 3-year DMFS was 100% in the 5-fraction group and 97.5% in the 4-fraction group (*P* = 0.058). Overall, CK-SBRT using 36 Gy in 4 fractions was well tolerated, with low rates of treatment-related toxicity and no statistically significant differences in oncologic outcomes between regimens. Longer follow-up is required to determine the long-term efficacy of this dose-escalation strategy.

## INTRODUCTION

Prostate cancer is the most commonly diagnosed cancer among men in the United States, with 288 300 new cases reported in 2023, ~70% of which were detected at a localized stage [1]. Due to the typically slow progression of prostate cancer, current guidelines recommend a risk-stratified management approach that considers the clinical stage, pathologic grade and patient life expectancy [2–4]. A variety of treatment options are available for managing localized prostate cancer (LPC), including active surveillance, radical prostatectomy and radiation therapy, all of which have reported 10-year overall survival rates of 100% [5].

Radiation therapy (RT) provides comparable efficacy to radical prostatectomy in treating LPC while avoiding the complications associated with surgery [6]. However, conventional fractionated RT usually requires a prolonged treatment course of 8–9 weeks to deliver a total dose of 74–80 Gy. Prostate cancer’s slow proliferation rate, characterized by a relatively low alpha-beta ratio [7] compared to that of surrounding normal tissues, suggests a greater sensitivity to higher doses per fraction, making hypofractionated regimens preferable for shorter treatment durations. Studies on moderate hypofractionation (2.5–4 Gy per fraction) have shown favorable outcomes with toxicity profiles similar to those of conventional fractionation [8–10].

Stereotactic body radiation therapy (SBRT), an intensified form of hypofractionation, is increasingly recognized as a viable option for LPC due to its ability to deliver high-dose radiation with precise targeting in 5 fractions or fewer. The CyberKnife system (CK, Accuray Inc., Sunnyvale, CA, USA) is specifically designed for SBRT. It utilizes a 6 MeV linear accelerator (LINAC) mounted on a robotic arm with six degrees of freedom. The wide range of beam directions allows for non-isocentric radiation delivery, enabling higher doses within the tumor while achieving excellent dose conformity. Additionally, it offers real-time tracking using fiducial markers placed within the prostate, which reduces the margins required for geometric uncertainties and minimizes radiation exposure to surrounding organs at risk. Several studies on CK-SBRT for LPC, which delivered a total dose of 35–36.25 Gy in 5 fractions, have reported excellent biochemical control rates with acceptable early and late toxicity profiles [11–14]. However, clinical data on prostate SBRT delivered in five or fewer fractions remain relatively limited [15], and studies exploring dose escalation using the CK platform are particularly scarce. Moreover, direct comparisons between different ultra-hypofractionated dose–fractionation schemes are lacking. In this context, the present study aims to evaluate the clinical outcomes of CK-based SBRT for localized prostate cancer, with a specific focus on the impact of two dose regimens: 35 Gy in 5 fractions and 36 Gy in 4 fractions.

## MATERIALS AND METHODS

Patients with LPC treated with SBRT from July 2011 through April 2021 at our medical center were consecutively enrolled in this study. The inclusion criteria were as follows: (1) age ≥ 20 years; (2) histologically confirmed prostate cancer; (3) no regional lymph node metastasis on abdominal pelvic imaging such as computed tomography (CT) or magnetic resonance imaging (MRI) and no distant metastasis on chest imaging (chest X-ray or CT) and bone scan; (4) no previous history of pelvic RT; (5) completion of planned SBRT. Patients with a follow-up period of ˂2 months were excluded. All patients were followed every 3 months during the first year after RT completion and every 6 months thereafter. Every follow-up visit included laboratory assessments of serum PSA and/or testosterone levels. Imaging (CT/MRI or prostate-specific membrane antigen positron emission tomography) was obtained for biochemical failure or rising PSA suspicious for recurrence. Patients lost to follow-up were censored at the last clinic visit. The medical records of included patients were retrospectively reviewed.

SBRT was delivered by CK G4 (CK, Accuray Inc., Sunnyvale, CA, USA). Gold fiducial markers were inserted within the prostate for real-time motion tracking during treatment. The prescription dose was either 35 Gy in 5 fractions or 36 Gy in 4 fractions every other day (EOD). Patients who started CK-SBRT before March 2019 underwent RT sessions of 5 fractions, while after March 2019, patients were treated with 4 fractions of CK-SBRT due to the reimbursement policy of the Health Insurance Review & Assessment Service of our country, which allowed SBRT to have fewer than 5 fractions. Given the low alpha–beta ratio of prostate cancer, the use of 36 Gy in 4 fractions corresponds to a higher biologically effective dose (BED_1.5_ = 211.6 Gy) than 35 Gy in 5 fractions (BED_1.5_ = 198.3 Gy); therefore, this regimen was adopted to achieve dose escalation while reducing the number of treatment fractions to four. RT plans were generated using the MultiPlan treatment planning system (V4.6; Accuray Inc., Madison, WI, USA) based on 1.25 mm thick, non-contrast CT images. The clinical target volume (CTV) was the prostate. To create a planning target volume (PTV), a 2 mm margin was added posteriorly, and a 3 mm margin was added in the other directions. The prescription dose encompassed 95% of the CTV. The dose constraints for the targets and organs at risk are detailed in Supplementary Table 1. Androgen deprivation therapy (ADT) was administered at the discretion of the treating urologist, taking into account not only the prostate cancer risk group but also the patient’s age, performance status and comorbidities. In most patients, ADT was initiated by the urologist before referral for radiotherapy and served as neoadjuvant treatment.

The primary endpoint was the incidence of grade 2 or greater toxicities. Genitourinary (GU) and gastrointestinal (GI) toxicities were defined by the Common Terminology Criteria for Adverse Events (CTCAE) Version 5.0. The secondary endpoints were biochemical recurrence-free survival rate (BCRFS), locoregional recurrence-free survival (LRRFS), distant metastasis-free survival (DMFS) and overall survival (OS) at 3 years. The biochemical recurrence (BCR) was defined as an increase of at least 2 ng/mL from the nadir PSA (Phoenix criteria) or the initiation of ADT. BCRFS was calculated from the last RT session to BCR or censoring at the last known follow-up. The locoregional recurrence was defined as any enlarged lymph node or novel mass within the prostate or pelvic region. The distant metastasis was defined as recurrence outside the pelvic region.

Baseline patient characteristics were compared between the treatment groups using the student’s t-test for continuous variables and the chi-square test or Fisher’s exact test for categorical variables. To account for potential bias, inverse probability of treatment weighting (IPTW) was utilized following consultation with the Department of Biostatistics to ensure appropriate methodological implementation. The balance of covariates between groups was evaluated using the standardized mean difference (SMD), where an SMD < 0.1 was considered to indicate negligible difference. Survival outcomes, including BCRFS, LRRFS and DMFS were analyzed using Kaplan–Meier curves and compared using the log-rank test. To identify predictors for toxicities and survival outcomes, univariate and multivariable analyses were performed. For multivariable analysis, variables with a *P*-value <0.1 in the univariate analysis were included in the initial model, and a backward elimination method was employed to identify independent predictors. Logistic regression analysis was employed to determine odds ratios (ORs) for chronic toxicity. Cox proportional hazards models were employed to determine hazard ratios (HRs) for both acute and chronic toxicities, as well as for survival outcomes, to account for the time-to-event nature of these endpoints. For the IPTW-weighted analysis, robust (sandwich) standard errors were applied to account for the weighting. All statistical analyses were performed using RStudio (Rstudio: Integrated Development for R. RStudio, PBC, Boston, MA). The present study protocol was reviewed and approved by the Institutional Review Board of our medical center (approval number: anonymized for review).

## RESULTS

A total of 249 patients were enrolled in this study. The baseline patient characteristics are shown in Table 1. The data show notable differences in the distribution of the National Comprehensive Cancer Network (NCCN) risk group and cT stage between the 5-fractions and 4-fractions groups (*P* = 0.007). Furthermore, the use of combined ADT and antithrombotic agents also differed significantly between the two groups (*P* = 0.001); the 4-fractions group had a higher percentage of patients receiving combined ADT (68.2%) compared to the 5-fractions group (47.1%). Except for four patients who received ADT concurrently with SBRT, the majority received ADT in the neoadjuvant setting. The mean duration of ADT was 19 months (range, 2–60) in the 4-fraction group and 19 months (range, 3–60) in the 5-fraction group. A greater proportion of the 5-fractions group were antithrombotic agent users (46.3%) relative to the 4-fractions group (24.6%). However, after IPTW application, all variables achieved a balance with a SMD < 0.1.

**Table 1:** Baseline patient and treatment characteristics before and after IPTW

		Before IPTW (*n* = 249)				After IPTW (n = 249)		
		No. (%)		*P*-value	SMD	No. (%)		SMD
Patient Characteristics		5 Fx (*n* = 123)	4 Fx (*n* = 126)			5 Fx (n = 123)	4 Fx (n = 126)	
Age	mean ± SD	72.76 ± 7.80	73.17 ± 6.71	0.663	0.055	73 ± 7.34	73 ± 6.89	0.002
ECOG performance status	0	64 (52.0)	74 (58.7)	0.455	0.142	72 (58.3)	75 (59.4)	0.02
	1	58 (47.2)	50 (39.7)			51 (41.1)	50 (39.6)	
	2	1 (0.8)	2 (1.6)			1 (0.6)	1 (1.01)	
NCCN risk group	Very low, low risk	11 (8.9)	18 (14.3)	0.007	0.413	18 (14.4)	16 (12.5)	0.045
	Intermediate risk	102 (82.9)	83 (65.9)			88 (71.2)	92 (73.4)	
	Very high, high risk	10 (8.2)	25 (19.8)			18 (14.4)	18 (14.1)	
cT stage	1	8 (6.5)	3 (2.4)	0.007	0.465	5 (4.1)	4 (3.3)	0.065
	2	109 (88.6)	103 (81.8)			104 (84.5)	108 (85.9)	
	3a	6 (4.9)	20 (15.9)			14 (11.4)	14 (10.8)	
Gleason score	≤7	118 (95.9)	118 (93.7)	0.418	0.103	116 (94.3)	119 (94.8)	0.024
	>7	5 (4.1)	8 (6.3)			7 (5.7)	7 (5.2)	
Prostate volume grouping (cc)	<50	90 (73.2)	102 (81.0)	0.144	0.186	106 (86.0)	109 (86.2)	0.004
	≥50	33 (26.8)	24 (19.0)			17 (14.0)	17 (13.8)	
Initial PSA grouping (ng/mL)	<10	107 (87.0)	108 (85.7)	0.769	0.037	48 (39.0)	51 (40.2)	0.023
	≥10	16 (13.0)	18 (14.3)			75 (61.0)	75 (59.8)	
Combined ADT	No	65 (52.9)	40 (31.8)	0.001	0.437	84 (68.1)	85 (67.5)	0.013
	Yes	58 (47.1)	86 (68.2)			39 (31.9)	41 (32.5)	
Antithrombotic agent user	No	66 (53.7)	95 (75.4)	0.001	0.467	98 (79.5)	99 (78.9)	0.015
	Yes	57 (46.3)	31 (24.6)			25 (20.5)	27 (21.1)	

Abbreviations: ADT = androgen deprivation therapy, cT stage = clinical T stage, ECOG = Eastern Cooperative Oncology Group, Fx = fractions, IPTW = inverse probability of treatment weighting, NCCN = National Comprehensive Cancer Network, No. = numbers, PSA = prostate-specific antigen, SMD = standardized mean difference.

**Fig. 1:**
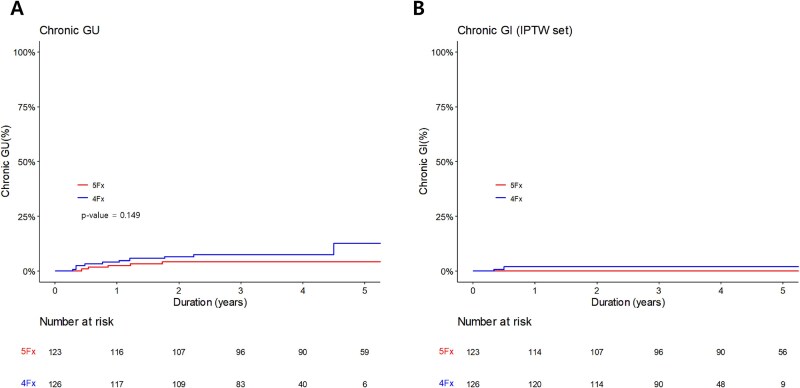
Cumulative incidence curves of chronic grade 2 genitourinary (GU) toxicities before IPTW **(A)** and after IPTW **(B)**.

The median follow-up time was 50 months (range, 2–136) for all patients. In the 5-fractions group, the median follow-up time was 59 months (range, 2–136), while it was 43 months (range, 2–64) in the 4-fractions group. Grade 2 GU toxicities occurred in 19 (7.6%) for acute (12 in 5-fractions and 7 in 4-fractions group) and 15 (6.0%) for chronic toxicity (5 in 5-fractions and 10 in 4-fractions group). No grade ≥ 3 GU toxicities were observed. The most common acute GU toxic event was nocturia (*n* = 13), followed by frequency (*n* = 9), urgency (*n* = 4), dysuria (*n* = 3) and urinary retention (*n* = 2). The most common chronic GU toxic event was nocturia (*n* = 9), followed by urinary frequency (*n* = 4), urgency (*n* = 4), dysuria (*n* = 2), erectile dysfunction (*n* = 2), hematuria (*n* = 1) and urinary retention (*n* = 1). There was no significant factor associated with grade 2 chronic GU toxicity (Table 2). Furthermore, in the IPTW cohort, RT fractionation did not significantly affect the occurrence of grade 2 chronic GU toxicity (OR, 2.17; 95% CI 0.71–6.66; *P* = 0.177). Figure  1 illustrates the cumulative incidence of GU toxicities before and after IPTW. There were no significant differences between two groups for cumulative incidence of GU toxicities.

**Table 2:** Univariate and multivariable analyses of grade 2 chronic genitourinary toxicities

		Univariate analysis			Multivariable analysis		
Patient characteristics		OR	95% CI	*P*-value	OR	95% CI	*P*-value
Age, years	≤ 75	1			1		
	> 75	0.44	0.14–1.42	0.169	0.40	0.11–1.25	0.139
NCCN risk group	≤ UI	1					
	> UI	1.58	0.42–5.90	0.498			
cT stage	≤ 2	1					
	> 2	1.35	0.29–6.33	0.707			
Gleason score	≤ 7	1					
	> 7	1.32	0.16–10.90	0.796			
Prostate volume (cc)	< 50	1					
	≥ 50	1.24	0.38–4.06	0.720			
Initial PSA (ng/mL)	< 10	1			1		
	≥ 10	2.47	0.74–8.27	0.141	3.25	0.83–11.01	0.067
RT fractionation	35 Gy/5 Fx	1			1		
	36 Gy/4 Fx	2.03	0.68–6.13	0.207	1.72	0.57–5.88	0.344
Combined ADT	No	1			1		
	Yes	2.09	0.65–6.75	0.219	2.07	0.65–7.90	0.242
Antithrombotic agent user	No	1					
	Yes	0.91	0.30–2.75	0.867			

Abbreviations: ADT = androgen deprivation therapy, CI = confidence interval, cT stage = clinical T stage, Fx = fractions, NCCN = National Comprehensive Cancer Network, OR = odds ratio, PSA = prostate-specific antigen, RT = radiotherapy, UI = unfavorable intermediate.

**Fig. 2:**
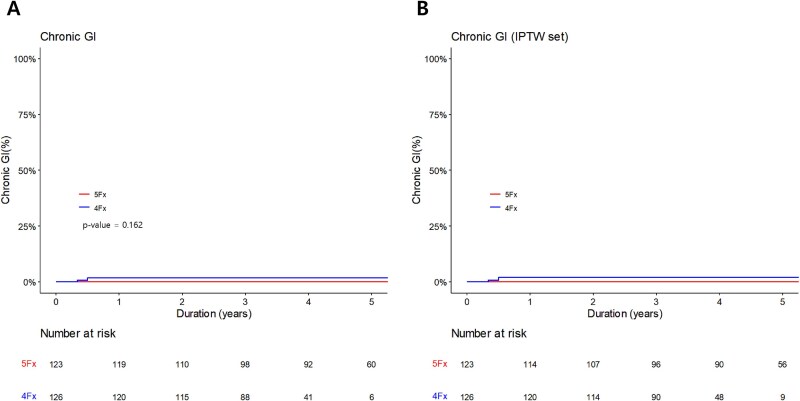
Cumulative incidence curves of chronic grade 2 gastrointestinal (GI) toxicities before IPTW **(A)** and after IPTW **(B)**.

Grade 2 GI toxicities were observed in three patients (1.2%) for acute GI toxicity and two patients (0.8%) for chronic GI toxicity, and all events occurred in 4-fractions group. Also, no grade ≥ 3 GI toxicities were observed. The most common acute GI toxic event was abdominal pain (*n* = 2), followed by bloating (*n* = 1), diarrhea (*n* = 1), fecal urgency (*n* = 1), loose stool (*n* = 1) and tenesmus (*n* = 1). Chronic GI toxic events included diarrhea (*n* = 1), fecal urgency (*n* = 1) and loose stool (*n* = 1). No rectal bleeding was observed. Figure  2 shows the cumulative incidence of GI toxicities before and after IPTW. There were no significant differences between two groups before IPTW. However, after IPTW, the *P*-value could not be calculated due to the absence of events in 5-fractions group.

**Fig. 3:**
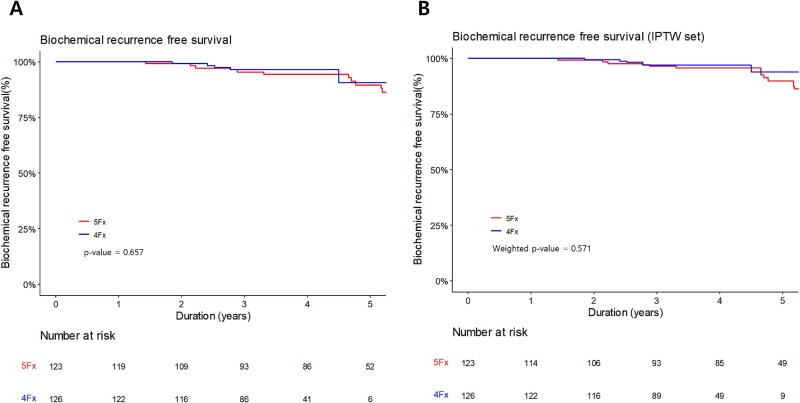
Kaplan Meier curves of biochemical recurrence before IPTW **(A)** and after IPTW **(B)**.

**Fig. 4:**
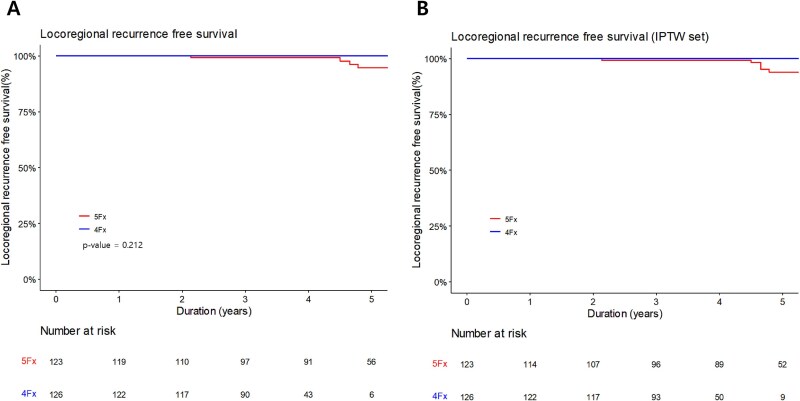
Kaplan Meier curves of locoregional recurrence before IPTW **(A)** and after IPTW **(B)**.

A total of 22 biochemical recurrences were identified, including 17 cases in the 5-fractions group and five cases in the 4-fractions group. The 3-year BCRFS was 95.3% and 96.4% in the 5-fractions and 4-fractions groups, respectively (*P* = 0.657; Fig.  3A). After IPTW, the 3-year BCRFS was 96.5% and 96.8%, respectively (*P* = 0.571; Fig.  3B). There were no significant differences between the two groups. Univariate and multivariate analyses revealed that the NCCN risk group was significantly associated with BCR (Table 3). Seven patients experienced locoregional recurrence, all of whom were in the 5-fractions group. Six patients experienced intraprostatic recurrence, while one patient experienced both intraprostatic and pelvic lymph node (right obturator lymph node) recurrence. The 3-year LRRFS was 99.1% and 100% in the 5-fractions group and 4-fractions group, respectively (*P* = 0.212; Fig.  4A). After IPTW, the 3-year LRRFS was 99.2% and 100%, respectively (Fig.  4B). Since there was no locoregional recurrence in 4-fractions group, after IPTW, the *P*-value could not be calculated. Univariate and multivariate analyses indicated that only initial PSA was significantly associated with locoregional recurrence (Supplementary Table 2).

**Table 3:** Univariate and multivariable analyses of biochemical recurrence

		Univariate analysis			Multivariable analysis		
Patient characteristics		HR	95% CI	*P*-value	HR	95% CI	*P*-value
Age, years	≤ 75	1					
	> 75	0.75	0.31–1.84	0.531			
NCCN risk group	≤ UI	1			1		
	> UI	3.35	1.19–9.38	0.022	3.56	1.26–10.07	0.017
cT stage	≤ 2	1					
	> 2	3.34	0.92–12.20	0.068			
Gleason score	≤ 7	1					
	> 7	2.00	0.46–8.60	0.354			
Prostate volume (cc)	< 50	1					
	≥ 50	1.84	0.77–4.39	0.170			
Initial PSA (ng/mL)	< 10	1					
	≥ 10	2.17	0.80–5.92	0.130			
RT fractionation	35 Gy/5 Fx	1			1		
	36 Gy/4 Fx	0.77	0.25–2.40	0.657	0.65	0.20–2.09	0.469
Combined ADT	No	1					
	Yes	0.44	0.18–1.09	0.077			

Abbreviations: ADT = androgen deprivation therapy, CI = confidence interval, cT stage = clinical T stage, Fx = fractions, HR = hazard ratio, NCCN = National Comprehensive Cancer Network, PSA = prostate-specific antigen, RT = radiotherapy, UI = unfavorable intermediate.

**Fig. 5:**
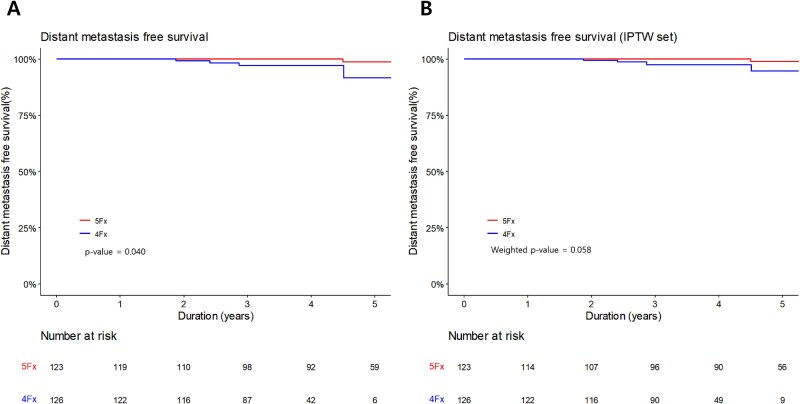
Kaplan Meier curves of distant metastasis before IPTW **(A)** and after IPTW **(B)**.

Six patients experienced distant metastases, with four cases occurring in the 4-fraction group. All four cases belonged to the high-risk group, and three of them did not receive ADT. The two patients who developed distant metastases in the 5-fraction group were both in the intermediate-risk group and had not received ADT. The 3-year DMFS was 100% and 97.2% in 5-fractions group and 4-fractions group, respectively (Fig.  5A). Additionally, a log-rank test demonstrated a significant difference between the two groups (*P* = 0.040). In contrast, after IPTW, the 3-year DMFS was 100% and 97.5%, respectively, which did not differ significantly (*P* = 0.058; Fig.  5B). Univariate and multivariable analyses indicated that NCCN risk group and prostate volume were statistically associated with distant metastasis (Supplementary Table 3). RT fractionation remained not significantly associated with distant metastasis in the multivariate analysis (*P* = 0.208). The 3-year OS for the entire cohort was 98.2%. Among the four deaths observed during follow-up, none were attributed to prostate cancer; all were due to other medical problems. No prostate cancer-specific mortality was identified.

## DISCUSSION

The present study confirmed the favorable clinical outcomes of the widely used 35 Gy in 5-fraction SBRT regimen for LPC and also demonstrated that dose escalation to 36 Gy in 4 fractions is feasible without substantially increasing toxicity. The use of SBRT for LPC is supported by two randomized trials [16–18] demonstrating its non-inferiority compared to conventional fractionation, as well as numerous prospective studies employing either CK or conventional linear accelerator platforms, all reporting favorable oncologic outcomes with acceptable toxicity [16–24] (Table 4). According to several meta-analyses, including various prospective clinical trials, the overall 5-year BCRFS was ~95%, with grade 2 or higher GU toxicity at 18%, and the GI toxicity at 10%. Grade 3 or higher late GU and GI toxicity rates were 2%, and 1%, respectively [25–28].

**Table 4:** Studies of stereotactic body radiation therapy using CK or conventional LINAC treatment system

Reference	Risk group	N	Method	Dose-fractionation	ADT (%)	BCRFS	Toxicity			
							GU, acute	GI, acute	GU, late	GI, late
Present study	LR 8.9% IR 83.0% HR 8.1%	123	CK	35 Gy/5fx, EOD	48.8%	95.3% at 3 years	G2 9.8% G3 0%	G2 0% G3 0%	G2 4.1% G3 0%	G2 0% G3 0%
	LR 14.3% IR 65.9% HR 19.8%	126	CK	36 Gy/4fx, EOD	69.8%	96.4% at 3 years	G2 5.6% G3 0%	G2 2.4% G3 0%	G2 7.9% G3 0%	G2 1.6% G3 0%
McBride *et al.* [19]	LR 100%	45	CK	36.25–37.5 Gy/5fx	Not allowed	97.7% at 3 years	G2 19%	G2 7%	G2 17% G3 2.2%	G2 7% G3 5%
Meier *et al.* [21]	LR 55.7% IR 44.3%	309	CK	40 Gy/5fx	1.3%	97.1% at 5 years	G2 26% G3 0%	G2 8.1% G3 0%	G2 12% G3 1.3%	G2 2% G3 0%
Fuller *et al.* [22]	LR 43% IR 57%	259	CK	38 Gy/4fx, daily	Not allowed	LR 100% at 5 years IR 88.5% at 5 years	G2 35.1% G3 1.1%	G2 6.9% G3 0%	G2 12.7% G3 1.9% G4 0.4%	G2 3.4% G3 0%
Krug *et al.* [23]	mostly IR	83	CK	35 Gy/5fx, daily or EOD	3.6%	98.8% at 1 year	G3 2.4%	G3 1.2%	G2 6.0% G3 2.4%	G2 0% G3 0%
Tree and van As *et al.* [16, 17]	LR 7.4% IR 92.6%	433	CK 41% LINAC 59%	36.25 Gy/5fx, daily or EOD	Not allowed	95.8% at 5 years	NR	NR	RTOG ≥G2 26.9% CTCAE ≥G2 42.5%	RTOG ≥G2 10.7% CTCAE ≥G2 18.8%
Widmark *et al.* [18]	IR 89% HR 11%	589	LINAC	42.7 Gy/7fx, EOD	Not allowed	83.9% at 5 years	G2 28% G3 6%	G2 8% G3 1%	G2 17.9% G3 4.2%	G2 9.5% G3 1.5%
Quon *et al.* [20]	LR 13.2% IR 86.8%	152	LINAC	40 Gy/5fx, EOD or weekly	4.6%	NR	EOD G2 31.6% G3 1.3% weekly G2 33.8 G3 2.7%	EOD G2 18.4% G3 0% weekly G2 10.8% G3 0%	EOD G2 45.3% G3 6.7% weekly G2 58.9% G3 2.7%	EOD G2 16% G3 1.3% weekly G2 23.3% G3 1.4% G4 1.4%
D’Agostino *et al.* [24]	LR 59% IR 41%	90	LINAC	35 Gy/5fx, EOD	13.3%	93.0% at 5 years	G2 32.2% G3 0%	G2 5.5% G3 0%	G2 3.3% G3 0%	G2 2.2% G3 0%

Abbreviations: LINAC = linear accelerator, ADT = androgen deprivation therapy, BCRFS = biochemical recurrence-free survival, GU = genitourinary, GI = gastrointestinal, LR = low risk, IR = intermediate risk, HR = high risk, CK = Cyberknife, fx = fractions, EOD = every other day, G = grade, NR = not reported.

Both the 4-fraction and 5-fraction groups in this study exhibited low toxicity rates, with no grade ≥3 events observed. This favorable profile is likely attributable to the distinct technical advantages of the CK system. Fiducial-based real-time tracking minimizes internal motion uncertainties, enabling reduced PTV margins, while the non-coplanar beam arrangement yields highly conformal dose distributions that can meet strict organ-at-risk (OAR) constraints without compromising target coverage. For the escalated dose regimen of 36 Gy in 4 fractions, we adopted constraints that were comparable to or more conservative than those employed in equivalent-dose SBRT regimens [20–22], assuming an α/β ratio of 3 for OARs. These constraints appear suitable for safe dose escalation without significantly increasing toxicity. The OAR constraints from the prospective trials are further described in Supplementary Table 1.

As with conventional fractionation, SBRT for LPC also exhibits a consistent dose–response relationship [29], and accordingly, several attempts have been made to escalate SBRT doses [30–32]. Based on the results of dose response and toxicity evaluation from 13 studies, increasing the total dose up to 40 Gy in 5 fractions appears to be well tolerated [33]. However, caution is needed when escalating toward 50 Gy [34]. In that study, among 91 patients who received 45–50 Gy, 7% and 6% experienced grade 3 or higher late GI and GU toxicities, respectively, including grade 4 cystitis requiring ureteroileal diversion, grade 4 rectal bleeding and six cases requiring colostomy. Strategies to enhance efficacy and expand the therapeutic window include hydrogel spacers [35], MRI-based image guidance [36] and simultaneous integrated boost approaches [37, 38].

The use of antithrombotic agents, in addition to dose-fractionation, has been associated with increased bleeding risk in both the GI and GU tracts [39, 40]. Musunuru *et al.* reported that, among patients treated with LINAC-based SBRT in five fractions, 47% of those on antithrombotic agents experienced grade 2 or higher hematochezia [40]. In contrast, our analysis found no significant association between antithrombotic use and bleeding risk. Although the 5-fraction group had a significantly higher rate of antithrombotic use than the 4-fraction group (46.3% vs. 24.6%, *P* = 0.001), bleeding events remained rare in both groups. These findings suggest that escalated-dose CK-SBRT can be safely administered to patients at risk of bleeding without the need for protective measures such as hydrogel spacers.

Oncologic outcomes were also promising, with the 3-year BCRFS reaching 95.3% in the 5-fraction group and 96.4% in the 4-fraction group, respectively. Local control and distant metastasis rates were similar between the groups. Although a slight increase in distant metastases was noted in the escalated-dose group, these events occurred primarily in high-risk patients who did not receive ADT. This observation, as supported by multivariate analysis, suggests that ADT may have had a more significant impact on distant metastasis than dose-fractionation. Despite concerns that strict OAR constraints might compromise target coverage, the comparable disease control rates indicate that the dose was effectively delivered to the target. A longer follow-up is needed to assess whether dose escalation provides significant long-term oncologic benefits, considering the high percentage of patients who received ADT due to the greater inclusion of unfavorable-intermediate and high-risk patients.

This study has several limitations. Its retrospective design introduces the possibility of incomplete documentation of toxicities, although all toxicities were systematically recorded at a single center. Additionally, baseline characteristics, such as risk group distribution and ADT use, differed significantly between the 35 Gy in 5 fractions and 36 Gy in 4 fractions groups, which may have influenced outcomes. Moreover, the median follow-up was shorter in the 4-fraction group (43 months) than in the 5-fraction group (59 months), restricting direct comparisons. Furthermore, because the study period spanned a decade and the four-fraction regimen was introduced later, temporal bias cannot be entirely excluded, although tracking and planning techniques remained consistent throughout the study period. Nevertheless, our findings highlight the potential benefits of dose escalation in fewer sessions while preserving a favorable toxicity profile.

## CONCLUSION

CK-SBRT using an escalated regimen of 36 Gy in 4 fractions was well tolerated and demonstrated favorable oncologic outcomes in patients with LPC, with acceptable toxicity profiles. Longer follow-up is required to determine the long-term benefits of this dose-escalation strategy.

## Supplementary Material

supplement_table_revision_final_rrag017

## Data Availability

Research data will be shared upon request to the corresponding author.
