# Supplementary material for: Clinical outcomes of CyberKnife stereotactic radiotherapy for localized prostate cancer: comparison of 35 Gy in 5 fractions and 36 Gy in 4 fractions
Source: J Radiat Res. 2026 Apr 14;67(3):402–11. doi: 10.1093/jrr/rrag017 (PMC13202314; doi:10.1093/jrr/rrag017)
Supplement: supplement_table_revision_final_rrag017 [file supplement_table_revision_final_rrag017.docx]

**Supplementary Table 1. Constraints for the targets and organs at risk in prospective studies**

| Reference | Dose-fractionation | Target | Rectum | Bladder | Urethra |
| --- | --- | --- | --- | --- | --- |
| Present study | 35 Gy/5fx,  EOD | CTV V35Gy ≥ 95%  PTV Dmin > 28 Gy | Dmax < 35 Gy  D1cc < 30.8 Gy | Dmax < 38.5 Gy  D1cc < 35.7 Gy | Dmax < 38.15 Gy |
|  | 36 Gy/4fx,  EOD | CTV V40Gy ≥ 95%  PTV Dmin > 28.8 Gy | Dmax < 35.28 Gy  D1cc < 30.96 Gy | Dmax < 38.52 Gy  D1cc < 36 Gy | Dmax < 38.16 Gy |
| McBride et al. [1] | 36.25-37.5 Gy /5fx | PTV V37.5Gy > 95% | V36Gy < 1 cc | V37.5Gy < 5 cc | V49Gy < 10% |
| Meier et al. [2] | 40 Gy/5fx | 36.25 Gy to PTV  40 Gy to CTV | V36Gy < 1cc | V37Gy < 5-10cc | V47Gy < 20% |
| Fuller et al. [3] | 38 Gy/4fx,  daily | PTV V38Gy > 95% | Outer wall Dmax < 38 Gy  Mucosa < 28.5 Gy | Dmax < 45.6 Gy  D10% < 41.8 Gy | Dmax < 45.6 Gy  D10% < 41.8 Gy  D50% < 39.9 Gy |
| Krug et al. [4] | 35 Gy/5fx,  daily or EOD | PTV V35Gy > 95% | Dmax < 38 Gy  V36Gy < 1 cc & 5%  V29Gy < 15 cc & 20%  V18Gy < 25 cc & 50% | Dmax < 38 Gy  V36Gy < 10 cc & 10%  V18Gy < 40% | Dmax < 44 Gy |
| Tree and van As et al. [5,6] | 36.25 Gy/5fx,  daily or EOD | PTV: V36.25Gy ≥ 95%  CTV: V40Gy ≥ 95% | V18.1Gy < 50%  V29Gy <20%  V36Gy < 1 cc | V18.1Gy < 40%  V37Gy < 10 cc | V42Gy < 50% |
| Widmark et al. [7] | 42.7 Gy/7fx,  EOD | PTV  V95%(40.6Gy) ≥ 95%  D99% ≥ 38.4 Gy | V38.4Gy ≤ 15%  V32Gy ≤ 35%  V28Gy ≤ 45% |  |  |
| Quon et al. [8] | 40 Gy/5fx,  EOD or weekly | CTV V100%(40Gy) ≥ 99%  PTV V95%(38Gy) ≥ 99% PTV V42Gy < 1 cc  PTV Dmax 42.8 Gy | V32Gy ≤ 15%  V28Gy ≤ 20% | V32Gy ≤ 15%  V28Gy ≤ 20% |  |
| D'Agostino et al. [9] | 35 Gy/5fx,  EOD | PTV: V95% ≥ 95%  CTV: V95% ≥ 99% | V32Gy < 5%  V28Gy < 10%  V18Gy < 35%  D1% < 35 Gy | D1% < 35 Gy |  |

Abbreviations: fx, fractions; EOD, every other day; PTV, planning target volume; CTV, clinical target volume; Dmin, minimum dose; Dmax, maximum dose; Dx (cc or %), dose received by the most irradiated volume (cubic centimeter or %); Vx (Gy or %), volume irradiated at X Gy or % of prescribed dose

**Supplementary Table 2.** **Univariate and multivariable analyses of locoregional recurrence**

|  |  | Univariate analysis | | | Multivariable analysis | | |
| --- | --- | --- | --- | --- | --- | --- | --- |
| Patient characteristics |  | HR | 95% CI | P-value | HR | 95% CI | P-value |
| Age, years | ≤ 75 | 1 |  |  |  |  |  |
|  | > 75 | 0.67 | 0.13-3.48 | 0.637 |  |  |  |
| NCCN risk group | ≤ UI | 1 |  |  |  |  |  |
|  | > UI | 2.14 | 0.25-18.24 | 0.487 |  |  |  |
| cT stage | ≤ 2 | 1 |  |  |  |  |  |
|  | > 2 | 2.01 | 0.06-62.91 | 0.690 |  |  |  |
| Gleason score | ≤ 7 | 1 |  |  |  |  |  |
|  | > 7 | 3.01 | 0.36-25.18 | 0.309 |  |  |  |
| Prostate volume (cc) | < 50 | 1 |  |  |  |  |  |
|  | ≥ 50 | 0.19 | 0.01-4.00 | 0.283 |  |  |  |
| Initial PSA (ng/mL) | < 10 | 1 |  |  | 1 |  |  |
|  | ≥ 10 | 6.28 | 1.40-28.25 | 0.017 | 6.34 | 1.41-28.44 | 0.016 |
| RT fractionation | 35 Gy/5 Fx | 1 |  |  | 1 |  |  |
|  | 36 Gy/4 Fx | 0.26 | 0.01-8.69 | 0.448 | 0.26 | 0.01-9.41 | 0.462 |
| Combined ADT | No | 1 |  |  |  |  |  |
|  | Yes | 0.46 | 0.09-2.39 | 0.357 |  |  |  |
| Abbreviations; ADT = androgen deprivation therapy, CI = confidence interval, cT stage = clinical T stage, Fx = fractions, HR = hazard ratio, NCCN = National Comprehensive Cancer Network, PSA = prostate-specific antigen, RT = radiotherapy, UI = unfavorable intermediate | | | | | | | |

**Supplementary Table 3. Univariate and multivariable analyses of distant metastasis**

| Patient characteristics | | Univariate analysis7 | | | Multivariable analysis | | |
| --- | --- | --- | --- | --- | --- | --- | --- |
|  |  | HR | 95% CI | P-value | HR | 95% CI | P-value |
| Age, years | ≤ 75 | 1 |  |  |  |  |  |
|  | > 75 | 0.83 | 0.15-4.59 | 0.834 |  |  |  |
| NCCN risk group | ≤ UI | 1 |  |  | 1 |  |  |
|  | > UI | 20.09 | 3.56-113.23 | 0.001 | 16.21 | 2.48-106.07 | 0.004 |
| cT stage | ≤ 2 | 1 |  |  |  |  |  |
|  | > 2 | 22.75 | 3.55-145.94 | 0.001 |  |  |  |
| Gleason score | ≤ 7 | 1 |  |  |  |  |  |
|  | > 7 | 3.67 | 0.43-31.46 | 0.236 |  |  |  |
| Prostate volume (cc) | < 50 | 1 |  |  | 1 |  |  |
|  | ≥ 50 | 6.22 | 1.14-33.96 | 0.035 | 6.38 | 1.09-37.30 | 0.040 |
| Initial PSA (ng/mL) | < 10 | 1 |  |  |  |  |  |
|  | ≥ 10 | 1.34 | 0.16-11.44 | 0.792 |  |  |  |
| RT fractionation | 35 Gy/5 Fx | 1 |  |  | 1 |  |  |
|  | 36 Gy/4 Fx | 8.33 | 0.85-82.22 | 0.069 | 4.69 | 0.42-52.11 | 0.208 |
| Combined ADT | No | 1 |  |  |  |  |  |
|  | Yes | 0.19 | 0.02-1.67 | 0.136 |  |  |  |
| Abbreviations; ADT = androgen deprivation therapy, CI = confidence interval, cT stage = clinical T stage, Fx = fractions, HR = hazard ratio, NCCN = National Comprehensive Cancer Network, PSA = prostate-specific antigen, RT = radiotherapy, UI = unfavorable intermediate | | | | | | | |
